# From Instagram to TikTok: How Social Media Fuels Eating Disorders, Anxiety, Depression, and Insomnia in Gen Z

**DOI:** 10.1192/j.eurpsy.2025.797

**Published:** 2025-08-26

**Authors:** A. H. I. Abu Shehab, A. B. Ciubară, S. L. Burlea, V. Doina Carina, M. Grigoraș, C.-F. Buicu, A. Ciubară

**Affiliations:** 1 Psychiatry, “Elisabeta Doamna” Psychiatry Hospital of Galati; 2 orthopaedics and traumatology, Faculty of Medicine and Pharmacy, “Dunărea de Jos” University, Galati; 3 Oral and maxillofacial surgery, 7University of Medicine and Pharmacy “Grigore T. Popa”, Iași; 4 Rheumatology; 5 Psychology, Faculty of Medicine and Pharmacy, “Dunărea de Jos” University, Galati; 6 Public health, “George Emil Palade” University of Medicine, Pharmacy, Science, and Technology, Targu Mures; 7 Psychiatry, Faculty of Medicine and Pharmacy, “Dunărea de Jos” University, Galati, Romania

## Abstract

**Introduction:**

Social media is now known as a vital aspect of the younger generation’s existence, significantly impacting their emotional and psychological growth. In contrast to prior generations, Generation Z has been raised in an environment of continuous digital connectivity, which poses distinct issues with body image, emotional health, and sleep quality. Platforms such as Instagram, TikTok, and Snapchat have been increasingly associated with mental health conditions, including eating disorders, anxiety, depression, and insomnia. Considering the ubiquity of these platforms, it is imperative for psychiatrists, public health practitioners, and educators to comprehend the mental health ramifications associated with social media participation.

**Objectives:**

This study attempts to examine the relationship between social media exposure and mental health disorders in Generation Z, specifically emphasizing eating disorders, anxiety, depression, and sleep disturbances.

**Methods:**

Relevant studies and existing research on social media usage patterns and their effects on mental health in Gen Z were reviewed. Attention was given to how different platforms contribute to the development of body dissatisfaction, social comparison, and sleep difficulties. The analysis included an exploration of platform-specific features and user behavior, focusing on Instagram’s image-centric nature, TikTok’s short-form, high-stimulation content, and Snapchat’s emphasis on transient communication.

**Results:**

The analysis indicated that Instagram, being predominantly a visual medium, exacerbates body dissatisfaction and increases the likelihood of eating disorders by consistently advocating unattainable beauty ideals and trends such as “thinspiration” and “fitspiration.” TikTok, although promoting mental health awareness, may also induce anxiety and attention deficits because to its intensely stimulating, rapid content. Snapchat’s design, that encourages continuous monitoring of temporary content, seems to exacerbate anxiety stemming from the fear of missing out (FOMO). Cyberbullying has surfaced as a substantial factor exacerbating mental health conditions across all platforms, particularly with anxiety and desperation, along with severe instances associated with suicidal ideation.

**Conclusions:**

Social media has a complex influence on the mental health of Generation Z, with each platform uniquely affecting conditions such as eating disorders, anxiety, depression, and insomnia. Instagram’s emphasis on aesthetics, TikTok’s rapid content consumption, and Snapchat’s addictive interaction present unique mental health issues. These findings underscore the necessity for medical professionals to integrate social media practices into their evaluations and therapies for Generation Z, while public health professionals and educators are encouraged to work together to promote better online habits.

**Disclosure of Interest:**

None Declared

